# The Impact of Allergic Rhinitis on Asthma and Its Effect on the Quality of Life of Asthmatic Patients

**DOI:** 10.7759/cureus.35714

**Published:** 2023-03-03

**Authors:** Shaima A Banjar, Raghad A Assiri, Ghada A Alshehri, Faris H Binyousef, Turki I Alaudah, Abdulmalik S Alawam, Abdulmalik M Aloriney

**Affiliations:** 1 College of Medicine, Al-Imam Mohammed Ibn Saud Islamic University, Riyadh, SAU; 2 Department of Family Medicine, Al-Imam Mohammed Ibn Saud Islamic University, Riyadh, SAU

**Keywords:** health-related quality of life, general practice < general medicine, asthma control, asthma, allergic rhinitis

## Abstract

Background

Allergic rhinitis (AR) and asthma are one of the most common diseases in the Kingdom of Saudi Arabia. Asthma and AR patients report significant reductions in their daily activities due to this condition. Therefore, measuring health-related quality of life (HRQOL) in adult asthmatic and AR patients and evaluating the use of allergic rhinitis treatment modalities to improve asthma control may help prevent future respiratory complications, improve patient quality of life, and reduce morbidity.

Methods

This cross-sectional observational study was conducted through an online self-administrated questionnaire distributed electronically on social media through “Survey Monkey” (http://www.surveymonkey.com) from April 2 to September 18, 2021. The study targeted adult patients with asthma and/or allergic rhinitis residing in the Riyadh region of Saudi Arabia. The study compared and evaluated HRQOL between three groups: asthmatic patients with concomitant AR, patients with asthma only, and patients with AR only.

Results

A total of 811 questionnaires were analyzed. Of those, 23.1% were diagnosed with asthma and 64% were diagnosed with allergic rhinitis; from those who were diagnosed with AR, 27.2% were asthmatics. A statistically significant association was observed between receiving AR medications and asthma control in respondents with intermittent AR (P < 0.001). However, no association was observed between asthma control and receiving medications for AR in respondents with persistent AR (P = 0.589). The average scores for all eight-item short-form (SF-8) QOL dimensions were lower in patients with combined asthma and AR than in patients with AR only and asthma only (P < 0.001).

Conclusions

This study suggested that AR was associated with more severe asthma and quality of life impairment.

## Introduction

Allergic rhinitis (AR) and asthma are one of the most common diseases in Saudi Arabia [1]. The estimated prevalence of AR in Saudi Arabia is 21.2% [2]. Asthma affects more than two million Saudis and accounts for 1.1% of the overall global estimates of “disability-adjusted life years” (DALYs)/100,000 for all causes [3,4]. Likewise, AR also caused a significant reduction in the daily activities of its patients [5]. Therefore, measuring the quality of life (QOL) in adult asthmatic and AR patients and evaluating the use of allergic rhinitis treatment modalities to improve asthma control may help prevent future respiratory complications, improve patient quality of life, and reduce morbidity. Various forms of QOL assessments have been used in previous studies, with the 36-item short-form survey (SF-36) being commonly used for assessing health-related quality of life (HRQOL). A short-form survey with eight scales (SF-8) has been developed as an excellent assessment tool for measuring HRQOL, especially in observational studies and studies with large populations [1]. SF-8 helps evaluate several important aspects of a patient’s health, including physical and social activity limitations, physical pain, psychological problems, and perceptions of general health [6]. This cross-sectional observational study was conducted in the Riyadh region of the Kingdom of Saudi Arabia to investigate the impact of allergic rhinitis on the quality of life of diagnosed and treated asthmatic patients and evaluate the role of therapeutic management of AR on asthma control. The health-related quality of life of asthmatic patients, AR patients, and those with rhinitis and concomitant asthma were also compared.

## Materials and methods

Study design

This is an observational descriptive cross-sectional study conducted through a self-administered questionnaire of adults residing in the Riyadh region of Saudi Arabia.

Study population, sample size, and technique

This study targets asthmatic adult patients residing in Riyadh. Randomly selected participants were selected through a questionnaire distributed online. According to recent studies, the prevalence of asthma in Riyadh, Saudi Arabia, is 18.2% [3]. Therefore, the sample size was assumed to be 730 patients based on the following sample size calculation formula: sample size (SS) = (Z2p (1 − p)) / C2, with a confidence interval (CI) of 95% and margin of error of 2.8%. In this study, the sample size was 811 patients with a confidence interval (CI) of 95% and margin of error of 2.66%.

Population selection

This research aims to demonstrate the known relationship between asthma and allergic rhinitis. Participants who fulfilled the following inclusion criteria were accepted in the research: residents of Riyadh, aged 16-66 years, clinically diagnosed with asthma for more than six months, and patients who consented to participate in the study. Moreover, participants who did not meet these criteria were excluded, as well as the following exclusion criteria: prior diagnosis of chronic obstructive pulmonary disease (COPD), pulmonary fibrosis, bronchitis, pneumonia, or COVID-19 for less than six months.

Data collection and questionnaire

Data collection began on April 2, 2021, and ended on September 18, 2021. Data collection was conducted via “Survey Monkey” (http://www.surveymonkey.com), utilizing a self-administrated online questionnaire through social media. Participants who refused to consent at the beginning of the questionnaire were immediately excluded from the research. The questionnaire contained five main sections (participants’ demographic, the Score For Allergic Rhinitis (SFAR), Allergic Rhinitis and Its Impact on Asthma (ARIA) guidelines, Asthma Control Test (ACT), and quality of life SF-8).

Score for Allergic Rhinitis (SFAR) Questionnaire

The SFAR is a self-administered tool used to diagnose allergic rhinitis. SFAR evaluates eight questions related to allergic rhinitis symptoms, time of occurrence during the year, triggers, allergy tests, and personal and family histories of allergy [7]. Patients with an SFAR score of ≥7 are considered to have AR, while patients with an SFAR score of <7 are considered to be free of AR [7]. The SFAR questionnaire Arabic version used in this study was validated [8].

Allergic Rhinitis and Its Impact on Asthma (ARIA) Questionnaire

The ARIA committee introduced a patient classification system to help the implementation of a stepwise approach to managing patients based on the time pattern of allergic rhinitis (AR) symptoms (intermittent versus persistent) and the severity (mild versus moderate/severe) [9]. The severity is classified based on “yes”/“no” answers to four questions.

Asthma Control Test (ACT) Questionnaire

The Asthma Control Test questionnaire is a tool that can be self-administered to identify those with poorly controlled asthma. The ACT questionnaire includes five Likert scale items, each on a scale from one to five, and it was validated in the Arabic edition [10]. The ACT items are scaled as follows: for symptoms and activities (5 = not at all and 1 = all the time) and for asthma control rating (5 = completely controlled and 1 = not controlled at all) [10].

The overall score for the ACT is calculated by summing the scores for the five items with a possible score ranging from 5 (poor control of asthma) to 25 (complete control of asthma). Higher scores reflect better asthma control. ACT cutoff points for uncontrolled and partly controlled asthma were ≤19 and ≤22, respectively [11,12]. Respondents whose asthma was reported as controlled or completely controlled were grouped when doing the analysis.

SF-8 Questionnaire

The SF-8 questionnaire was used in the current study to assess the quality of life of the included respondents (Table 1). The SF-8 includes eight single-item scales in addition to two summary components: physical component summary (PCS) and mental component summary (MCS) [6]. These two summary measures can be calculated by weighting SF-8 items using a norm-based scoring method [6]. Higher scores for the individual items and summary components indicate better health (mean value: 50). The SF-8 includes the following eight items and their respective response categories: general health (GH), physical functioning (PF), role physical or difficulties with daily work because of physical pain (PP), bodily pain (BP), vitality (VT), social functioning (SF), mental health or emotional problems (MH), and role emotional or absence from daily activities because of emotional problems (RE) [6].

**Table 1:** SF-8 questionnaire SF-8: eight-item short-form survey

Overall, how would you rate your health during the past four weeks?					
Excellent 	Very good 	Good 	Fair 	Poor 	Very poor 
During the past four weeks, how much did physical health problems limit your usual physical activities (such as walking or climbing stairs)?					
Not at all 	Very little 	Somewhat 	Quite a lot 	Could not do physical activities 	
During the past four weeks, how much difficulty did you have doing your daily work, both at home and away from home, because of your physical health?					
None at all 	A little bit 	Some 	Quite a lot 	Could not do daily work 	
How much bodily pain have you had during the past four weeks?					
None 	Very mild 	Mild 	Moderate 	Severe 	Very severe
During the past four weeks, how much energy did you have?					
Very much	Quite a lot	Some 	A little 	None 	
During the past four weeks, how much did your physical health or emotional problems limit your usual social activities with family or friends?					
Not at all 	Very little 	Somewhat 	Quite a lot 	Could not do social activities 	
During the past four weeks, how much have you been bothered by emotional problems (such as feeling anxious, depressed, or irritable)?					
Not at all	Slightly 	Moderately 	Quite a lot 	Extremely 	
During the past four weeks, how much did personal or emotional problems keep you from doing your usual work, school, or other daily activities?					
Not at all	Very little 	Somewhat 	Quite a lot 	Could not do social activities 	

The SF-8 was scored by assigning the mean SF-36 scale score from the 2020 Saudi population [13] to each response category of the SF-8 measuring the same concept and then weighting each SF-8 item to compute composite physical (PCS-8) and mental (MCS-8) summary scales. Principal component analysis (PCA) was used to derive the summary component measures to improve their ability to discriminate between mental and physical health outcomes [14]. There are published online algorithms that can be used to score the SF-8 [15].

The ARIA and SF-8 questionnaires were translated into Arabic, the official language of Saudi Arabia, by a bilingual Saudi physician. Then, it was back-translated into English by another translator who does not know the English version. Forward translation into Arabic was conducted. The Arabic version was then pilot tested for understanding in a small patient group before it was used.

Statistical analysis

Responses were collected and exported to an Excel sheet (Microsoft Corp., Redmond, WA, USA) for further analysis. Statistical analysis was performed using Rv 3.6.3. Counts and percentages were used to summarize the distribution of categorical variables. The mean ± standard deviation (SD) was used for continuous variables. The chi-square test of independence was used to assess the association between categorical variables. Unpaired t-test and one-way analysis of variance (ANOVA) were used to compare the distribution of continuous variables between groups with two and more than two levels, respectively. For one-way ANOVA, post hoc comparisons were performed using the unpaired t-test with correction for false discovery rate. Hypothesis testing was performed at a 5% level of significance.

Ethical approval

All participants were provided with sufficient information regarding the study’s aims. Personal data remain confidential and are used for research purposes only. A scientific committee and institutional review board (IRB) in Imam Mohammed Ibn Saud Islamic University (IMSIU) was set up to validate the scientific quality of the project and the relevance of the objectives and methodology and to control the progress of the study. This study was conducted in accordance with the Helsinki Declaration and was approved by local legal authorities including the standing committee for research ethics on living creatures (approval number: 3802022).

The confidentiality of personal information is the most important consideration in this study. All information collected was protected, and patient anonymity was ensured. No identifying information was stored with the data.

## Results

The study questionnaire was completed by 878 respondents between April 2 and September 18, 2021. A total of 67 (8.3%) participants were excluded from the analyzed population due to incompatibility with the inclusion criteria, i.e., disagreeing to participate in the study (n = 2), not living in Riyadh city or any of its provinces (n = 23), age < 16 years old (n = 3), has chronic obstructive pulmonary disease (COPD), pulmonary fibrosis, bronchitis, or pneumonia (n = 8), and been diagnosed with COVID-19 since less than six months (n = 31). Overall, the study analyzed 811 questionnaires.

Respondents’ characteristics

A total of 811 questionnaires were analyzed. Of these, 95.6% were living in Riyadh city and 4.4% were living in one of Riyadh’s provinces. Males and females represented 43% and 57% of the study sample, respectively. Respondents aged 16-31 years old represented 69.2% of the study sample, while respondents aged 32-47, 48-63, and 64 or more years represented 15.8%, 14.3%, and 0.7% of the study sample, respectively. The average body mass index (BMI) was 26.4 ± 6.5 kg/m^2^, with 45.1% overweight respondents. Overweight and obese respondents represented 28.5% and 21.7% of the study sample, respectively. Two-thirds of the respondents had a bachelor’s degree or higher (64.9%), and one-third (34%) completed only high school. The majority of the respondents were non-smokers (81.8%), and 13.8% were current smokers. One-quarter of the respondents had asthma (23.1%), and a similar number had allergic rhinitis (23.8%). The demographic characteristics of the patients are presented in Table 2. Of those with asthma, 71.1% have been diagnosed for more than five years, 19.8% have been diagnosed for less than two years, and 9.1% have been diagnosed for less than five years and more than two years.

**Table 2:** Demographic and clinical characteristics of respondents The percentages are calculated on the number of available data. SD: standard deviation, BMI: body mass index

Demographic and clinical characteristics	Number (%)
Sex	
Female	462 (57%)
Male	349 (43%)
Age (years)	
16-31	561 (69.2%)
32-47	128 (15.8%)
48-63	116 (14.3%)
64-70	6 (0.7%)
BMI (mean ± SD)	26.4 (6.5)
BMI category	
Underweight	38 (4.7%)
Normal	366 (45.1%)
Overweight	231 (28.5%)
Obese	176 (21.7%)
Education	
Intermediate school	9 (1.1%)
High school	276 (34%)
College and above	526 (64.9%)
Smoking status	
Never smoked	663 (81.8%)
Ex-smoker	36 (4.4%)
Current smoker	112 (13.8%)
Comorbidities	
None	168 (20.7%)
Allergic rhinitis	519 (64%)
Asthma	187 (23.1%)
Cardiovascular disease	7 (0.9%)
Diabetes	40 (4.9%)
Dyslipidemia	20 (2.5%)
Gastroesophageal reflux	45 (5.6%)
Hypertension	38 (4.7%)
Thyroid disorder	36 (4.4%)

Allergic rhinitis and asthma

Asthma was present in 23.1% of respondents. The frequency of AR in asthmatic patients was 75.4% (95%CI: 71.7%-78.9%) in 141 out of the 187 respondents. Of those with asthma, allergic rhinitis was intermittent for 78% of patients (95%CI: 76.9%-79.1%) and persistent for 22% of patients (95%CI: 20.9%-23.1%). However, AR was mild for 22.7% of patients (95%CI: 21.6%-23.8%) and moderate/severe for 77.3% of patients (95%CI: 76.2%-78.4%).

Relationships between quality of life, asthma, and allergic rhinitis

The exploratory factor analysis for SF-8 is shown in Table 3. The average scores for all SF-8 dimensions except VT (P = 0.24) and MH (P = 0.103) differed significantly between patients with various asthma control levels. The average scores for the PCS and MCS summary components were also significantly different between the three groups (P < 0.001 and 0.001, respectively). In particular, the average scores for all SF-8 dimensions were significantly lower in patients with uncontrolled asthma compared to the remaining two groups. Moreover, there was an increasing trend in the average SF-8 score with the increase in the level of asthma control. This was observed for the GH, PF, PP, SF, MH, RE, PCS, and MCS domains. The average levels of the BP and VT domains were similar in patients with uncontrolled and partially controlled asthma and were lower in the formed two groups than in patients with controlled asthma (Table 4).

**Table 3:** Exploratory factor analysis for SF-8 Numbers between brackets represent the weights from PCA that were used to compute PCS and MCS. Exploratory factor analysis was performed using Varimax rotation. SF-8: eight-item short-form survey, SF-36: 36-item short-form survey, PCS: physical component summary, MCS: mental component summary, GH: general health, PF: physical functioning, PP: role physical, BP: bodily pain, VT: vitality, SF: social functioning, RE: role emotional, MH: mental health, PCA: principal component analysis

	PCS	MCS	Saudi norm SF-36
GH	0.46 (0.62)	0.24	48.73
PF	0.90 (0.9)	0.16	46.42
PP	0.88 (0.89)	0.19	50.09
BP	0.70 (0.77)	0.36	50.58
VT	0.25 (0.35)	0.19	49.79
SF	0.40	0.54 (0.66)	46.18
RE	0.18	0.88 (0.89)	43.18
MH	0.23	0.79 (0.89)	47.27
Cronbach’s α	0.79	0.82	

**Table 4:** Quality of life (SF-8) based on asthma control and the severity of AR Data were summarized using mean and standard deviation. Statistical analysis was performed using one-way ANOVA. SF-8: eight-item short-form survey, AR: allergic rhinitis, ANOVA: analysis of variance, SD: standard deviation, GH: general health, PF: physical functioning, PP: role physical, BP: bodily pain, VT: vitality, SF: social functioning, RE: role emotional, MH: mental health, PCS: physical component summary, MCS: mental component summary

	Asthma (mean ± SD)					Allergic rhinitis (mean ± SD)					
	All	Uncontrolled	Partially controlled	Controlled	P value	All	Intermittent mild	Intermittent moderate-severe	Persistent mild	Persistent moderate-severe	P value
GH	66.6 (14.9)	52.6 (15.3)	62.3 (11.8)	72.4 (12.6)	<0.001	69.6 (15.3)	74.9 (11.3)	67.8 (15.7)	78.3 (9.70)	60.3 (16.5)	<0.001
PF	62.0 (16.6)	45.5 (17.5)	55.6 (17.1)	69.3 (10.6)	<0.001	65.0 (16.3)	70.4 (11.1)	62.2 (16.8)	75.8 (6.91)	57.6 (19.8)	<0.001
PP	67.2 (18.2)	54.3 (21.2)	62.6 (17.7)	72.8 (14.9)	<0.001	71.4 (17.1)	77.3 (10.4)	68.3 (18.5)	82.6 (3.73)	63.8 (19.7)	<0.001
BP	67.8 (19.2)	57.4 (15.8)	59.1 (18.4)	74.2 (17.8)	<0.001	68.9 (19.7)	76.7 (15.5)	66.3 (20.0)	78.7 (14.6)	57.8 (20.3)	<0.001
VT	47.4 (15.4)	44.1 (9.98)	46.0 (15.5)	48.9 (16.6)	0.240	51.1 (14.7)	57.1 (16.4)	50.1 (12.5)	47.2 (12.7)	48.4 (17.6)	<0.001
SF	57.3 (19.1)	46.7 (18.9)	56.0 (14.9)	60.9 (19.5)	0.001	55.4 (18.4)	60.8 (17.1)	50.4 (18.3)	63.4 (18.3)	56.9 (15.9)	<0.001
MH	44.3 (17.9)	38.2 (15.3)	46.1 (17.7)	45.4 (18.4)	0.103	47.1 (19.2)	55.1 (16.8)	42.4 (18.6)	52.4 (22.0)	46.2 (17.2)	<0.001
RE	55.5 (18.7)	43.8 (16.9)	58.7 (15.7)	57.6 (19.1)	<0.001	57.6 (19.7)	64.7 (17.6)	53.0 (19.8)	64.4 (19.2)	56.5 (18.5)	<0.001
PCS	65.3 (13.8)	52.4 (13.4)	59.6 (12.1)	71.2 (10.8)	<0.001	68.3 (13.8)	74.5 (8.76)	65.8 (14.2)	77.3 (5.45)	60.0 (15.4)	<0.001
MCS	51.9 (15.8)	42.6 (14.0)	53.4 (14.2)	54.0 (16.0)	0.001	53.2 (16.7)	60.1 (14.8)	48.5 (16.1)	59.7 (17.6)	52.8 (15.4)	<0.001

Regarding the association between the severity of AR and quality of life, the SF-8 scores for all items and the summary components were significantly different between the four severity grades of AR. It was noted that the average levels for all SF-8 items were lower in patients with persistent AR than in patients with intermittent AR. Moreover, the average levels were lower in patients with moderate-severe AR than in patients with mild AR. The same findings were observed for the SF-8 summary components (Table 5).

**Table 5:** Association between disease and SF-8 score Data were summarized using mean and standard deviation. Statistical analysis was performed using one-way ANOVA. Post hoc pairwise comparisons were performed using an unpaired t-test. SF-8: eight-item short-form survey, ANOVA: analysis of variance, AR: allergic rhinitis, GH: general health, PF: physical functioning, PP: role physical, BP: bodily pain, VT: vitality, SF: social functioning, RE: role emotional, MH: mental health, PCS: physical component summary, MCS: mental component summary

	AR (n = 326)	Asthma (n = 46)	Asthma + AR (n= 141)	P (overall)	AR versus asthma	AR versus combined	Asthma versus combined
GH	71.2 (14.7)	69.0 (11.0)	65.9 (16.0)	0.002	0.628	0.001	0.423
PF	67.7 (15.7)	71.6 (14.4)	58.8 (16.0)	<0.001	0.253	<0.001	<0.001
PP	74.9 (15.3)	79.5 (11.2)	63.2 (18.3)	<0.001	0.163	<0.001	<0.001
BP	71.5 (19.5)	82.6 (8.97)	62.9 (19.2)	<0.001	<0.001	<0.001	<0.001
VT	52.6 (14.2)	47.3 (16.4)	47.4 (15.2)	0.001	0.053	0.001	0.997
SF	56.2 (18.1)	69.3 (14.1)	53.4 (18.9)	<0.001	<0.001	0.259	<0.001
RE	49.0 (18.9)	49.4 (12.0)	42.7 (19.2)	0.002	0.988	0.002	0.079
MH	59.5 (19.4)	63.0 (12.0)	53.1 (19.8)	0.001	0.475	0.002	0.006
PCS	70.9 (13.0)	74.5 (9.53)	62.2 (13.6)	<0.001	0.175	<0.001	<0.001
MCS	54.8 (16.6)	59.8 (10.4)	49.4 (16.4)	<0.001	0.124	0.002	<0.001

Results showed that the average scores for all SF-8 items and the summary components differed significantly between the three groups. The average levels for all SF-8 items were not significantly different between patients with AR and patients with asthma except for the BP, VT, and SF, which were higher in patients with asthma only than in patients with AR. The average scores were lower in patients with combined asthma and AR than in patients with AR only and asthma only. The only exceptions were the SF, GH, and VT domains. The average SF score was not significantly different between patients with AR only and combined AR and asthma (P = 0.259). The average GH and VT scores were not significantly different between patients with asthma only and patients with combined asthma and AR (P = 0.423 and 0.997, respectively).

The impact of allergic rhinitis treatment on asthma control level

Analysis showed that allergic rhinitis status (positive versus negative) was significantly associated with asthma control (P = 0.001). The proportion of patients with allergic rhinitis was significantly lower in respondents with controlled asthma (65.8%) than in respondents with uncontrolled (84.4%) or partially controlled (93.2%) asthma. When the analysis was excluded to respondents with intermittent AR (n = 110), whether mild or moderate-persistent, a statistically significant association was observed between receiving AR medications and asthma control (P < 0.001). Fewer respondents with controlled asthma reported receiving medications for AR (31.8%) than respondents with partially controlled (73.3%) or uncontrolled (78.6%) asthma. No association was observed between asthma control and receiving medications for AR in respondents with persistent AR (P = 0.589). However, only a few respondents had asthma and persistent AR (n = 31), which may have affected the power to detect a statistically significant effect size (Table 6).

**Table 6:** AR treatment and asthma control level Counts and percentages were used to summarize the data. A chi-square test of independence was used for statistical analysis. AR: allergic rhinitis

AR	Asthma control				P value
	Overall (number (%))	Uncontrolled (number (%))	Partially controlled (number (%))	Controlled (number (%))	
Without AR	46 (24.6%)	5 (15.6%)	3 (6.82%)	38 (34.2%)	0.001
With AR	141 (75.4%)	27 (84.4%)	41 (93.2%)	73 (65.8%)	
Intermittent					
Not on treatment	56 (50.9%)	3 (21.4%)	8 (26.7%)	45 (68.2%)	<0.001
On treatment	54 (49.1%)	11 (78.6%)	22 (73.3%)	21 (31.8%)	
Persistent					
Not on treatment	14 (45.2%)	6 (46.2%)	6 (54.5%)	2 (28.6%)	0.589
On treatment	17 (54.8%)	7 (53.8%)	5 (45.5%)	5 (71.4%)	

## Discussion

Our study aimed to investigate the impact of the presence of allergic rhinitis (AR) and its management on the quality of life of diagnosed and treated asthmatic patients with different levels of control. The majority of our respondents had good health habits, and 81.8% of the participants were non-smokers. However, overweight and obese participants represented 28.5% and 21.7% of the sample, respectively.

Our results showed that while 23.1% of the participants were diagnosed with asthma, the prevalence of AR in asthmatic patients was 75.4%, consistent with the estimated prevalence reported in the literature, whereas the prevalence of AR exceed 50% in patients with asthma in the United States and up to 100% prevalence in patients with allergic asthma in Europe [16]. A study conducted by Magnan et al. reported a prevalence of AR in asthmatic patients of 55.2% in the French population [17]. Several risk factors for the development of asthma and AR have been identified, including positive family history, elevated serum IgE levels greater than 100 IU/mL, or positive allergy skin prick test [18]. AR and asthma are correlated, sharing similar epidemiology, pathophysiological mechanisms, and therapeutic approaches [19]. In patients with AR, subclinical inflammatory changes and eosinophilia can be detected in the lower airways, resulting in bronchial hyper-reactivity and a reduced forced expiratory flow and pulmonary volume, suggesting the importance of managing asthma in patients with persistent allergic rhinitis [20,21]. Of adults with AR, 60% reported nasal congestion as the most common symptom, followed by rhinorrhea, sneezing, and nasal itch, which consequently may contribute to a substantial burden on patients’ quality of life, sleep quality, and work productivity [22]. A cohort study consisting of 49 patients with persistent AR conducted to evaluate the impact of AR on smell detection demonstrated that individuals with moderate or severe persistent AR showed a significant reduction in smell detection compared to healthy controls and that 67% of participants reported hyposmia [23]. Other comorbidities may be associated with AR, including migraine headaches (17% prevalence in those diagnosed with AR compared to 9% in nonallergic adults), skin rashes (10%-15% in AR patients versus 3% in nonallergic adults), and earaches and gastroesophageal reflux [24].

This study demonstrated a significant difference in mean scores for all SF-8 sections in patients with various asthma control levels, except for individuals with uncontrolled asthma, where they demonstrated significantly lower scores on all aspects of vitality and mental health. These scores corresponded with the study by González-Freire et al., where they found that poor asthma control was associated with a worse quality of life in all dimensions except mental health [25]. Likewise, similar results were concluded in a national study based in France, in which the findings revealed that asthma and AR did not impair the quality of life in terms of mental disability and well-being [26]. Another recently published nationwide database study revealed a significant difference between the asthma/AR group and the control group in the presence of strong psychological stress, psychiatric consultations, and depression with increased risk in asthma patients (odds ratio (OR): 3.03, 95%CI: 1.29-7.09) [27]. Moreover, a cross-sectional study conducted among the French population to evaluate the quality of life of asthmatic patients affected by AR revealed that the severity of AR significantly increased the severity of asthma [17] and was associated with worse asthma control and poor quality of life [17].

In terms of psychological disturbance, there is a strong established association between depression and asthma. In a cross-sectional study among 164 asthmatic patients, results showed that 54.3% and 50.6% of patients had anxiety and depression, respectively [28]. In a cohort study that aimed to assess the impact of anxiety and depression on asthmatic patients, the authors found that asthma exacerbation was associated with the level of depression, especially among females [29]. Additionally, according to a systematic review and meta-analysis of 24 primary studies published in 2021, AR is associated with an increased risk of depression and anxiety [30]. There are several mechanisms that might explain why allergic reactions may negatively affect cognitive function or trigger the immune system and cytokines resulting in nasal obstruction and the subsequent effect on the quality of sleep [31]. Like other allergic diseases, atopic dermatitis may have a negative impact on quality of life. In Korea, a nationwide database of the Korean population including 37,578 adults’ quality of life was assessed using EuroQoL (EQ) five-dimension questionnaire and EQ-visual analog scale score; the results revealed that poor quality of life was significantly associated with atopic dermatitis [32].

Regarding the management of asthma and AR, routine surveillance to detect rhinitis in patients with asthma has been recommended in collaboration with the World Health Organization (WHO) [33]. Additionally, treatment of AR with intranasal corticosteroids and montelukast improves sleep disturbance and productivity, thereby improving quality of life [34]. According to the 2016 guidelines on Allergic Rhinitis and Its Impact on Asthma (ARIA), appropriate treatment of AR using oral/intranasal H1-antihistamines, intranasal corticosteroids, and leukotriene receptor antagonists either alone or in combination might improve patients’ quality of life and work productivity [35].

This study had several limitations. This is a cross-sectional study, which could not predict causal relationships. Our results relied on self-reported data without accurate diagnostic assessment of asthma and AR. We were also unable to analyze the confounding effect in our analysis, including gender, BMI, smoking, and other comorbidities. However, our large-scale epidemiological study assessed the quality of life using the standardized questionnaire with subanalysis according to disease severity.

## Conclusions

This study highlighted the strong association between AR severity and asthma severity and its negative impact on quality of life. Among the different levels of asthma control, the mean scores for the PCS and MCS summary components and all SF-8 dimensions were significantly different, except for VT and MH. Lower scores were observed in patients with uncontrolled asthma and patients with persistent AR. There was an increasing trend in the mean SF-8 score with the increase in the level of asthma control. Additionally, a statistically significant association was observed between AR medication use and asthma control. Poor asthma control and AR are related conditions that can impact the quality of life. We recommend routine screening for AR and appropriate management in asthmatic patients, particularly those with severe and uncontrolled levels of asthma, with a view to managing mental health problems and improving quality of life.
